# Attentional Function in Patients With Amyotrophic Lateral Sclerosis is Moderated by Age and Education

**DOI:** 10.1002/brb3.71511

**Published:** 2026-06-03

**Authors:** Maher Zoubi, Patrick Weydt, Xenia Kobeleva

**Affiliations:** ^1^ Center For Neurology University Hospital Bonn Bonn Germany; ^2^ DZNE Bonn Bonn Germany; ^3^ Computational Neurology Group, Medical Faculty Ruhr University Bochum Bochum Germany

**Keywords:** amyotrophic lateral sclerosis, attentional function, cognitive reserve, executive function, neuropsychology

## Abstract

**Purpose:**

Besides motor brain regions, amyotrophic lateral sclerosis (ALS) affects non‐motor regions such as front temporal regions, affecting various cognitive domains.

**Method:**

We performed a behavioral study using the attention network test (ANT) to examine two components of attention (alerting, executive condition) and two degrees of difficulty (conflict condition) in 27 patients with ALS with no reported symptoms suggestive of cognitive impairment and 26 matched control participants.

**Findings:**

Using a modified ANT that accounted for ALS‐induced motor impairment by focusing on relative reaction times, we could demonstrate its feasibility even in severely paralyzed patients. Relative reaction time differences were comparable to controls, demonstrating the task's ability to correct for motor bias. When focusing on relative reaction times, in both groups we found intact executive and conflict effects. Furthermore, ALS patients had comparable task accuracies when reacting to congruent and incongruent easy targets. However, the task accuracy of ALS patients was significantly lower compared to controls when reacting to the incongruent hard target. This effect was enhanced by the interaction effect of ALS diagnosis and age.

**Conclusion:**

Our results suggest a significant interaction between age and ALS pathology, potentially leading to a breakdown of cognitive resources at higher levels of executive demand. We hypothesize that subclinical executive vulnerability in ALS patients becomes apparent when additional detrimental factors, such as aging, are present in patients. While we did not test co‐pathologies in our cohort, co‐occurring neurodegenerative or vascular processes might have contributed to this result. Our findings highlight the importance of cognitive screening for ALS patients above 60 years, even in the absence of subjective and collateral history of cognitive impairment.

## 1 Introduction

Amyotrophic lateral sclerosis (ALS) is a devastating neurodegenerative disease that affects motor and non‐motor CNS regions of the central nervous system (Neumann et al. 2006; DeJesus‐Hernandez et al. 2011; Renton et al. 2011; Abrahams et al. 2005; Mohammadi et al. 2015; Stoppel et al. 2014; Brettschneider et al. 2013). Due to the involvement of non‐motor brain regions such as frontotemporal areas (Strong et al. 1999), cognitive impairment is common in ALS, with up to 55% of patients affected (Phukan et al. 2012; Ringholz et al. 2005; Montuschi et al. 2015; Chiò et al. 2019; Rippon et al. 2006), depending on the exact definition of cognitive impairment. Front temporal neurodegeneration has been mainly associated with impairment in cognitive domains such as verbal fluency and executive function (Phukan et al. 2012; Beeldman et al. 2016). Although attentional function heavily depends on frontal lobe function (Vergel Hernández et al. 2021), it has been much less studied, especially in interaction with executive dysfunction. However, quantifying potential attentional impairment in ALS is crucial, as attention serves as the foundational cognitive process for a wide array of higher‐order functions and activities of daily living.

Due to the limited number of studies on attention in ALS, existing studies present conflicting findings regarding attentional dysfunction in ALS (Mohammadi et al. 2015; Stoppel et al. 2014; Abrahams et al. 2014; Castelnovo et al. 2020; Pinkhardt et al. 2008; Kobeleva et al. 2021; Thorns et al. 2010; Christidi et al. 2012; Goldstein et al. 2011). Some studies indicate a possible attention deficit in ALS evidenced by reduced accuracy (Kobeleva et al. 2021; Thorns et al. 2010; Christidi et al. 2012; Goldstein et al. 2011; McMackin et al. 2020) and prolonged reaction times (Mohammadi et al. 2015; Kobeleva et al. 2021; Thorns et al. 2010) in behavioral tasks. However, other studies have found intact attentional function in ALS patients in behavioral tasks (Stoppel et al. 2014), but indicate enhanced brain activity in mainly prefrontal brain areas as a possible sign of compensatory brain activity (Pinkhardt et al. 2008; McMackin et al. 2021).

Motor impairment in ALS, leading to prolonged reaction times independently of the cognitive task (Kobeleva et al. 2021; Thorns et al. 2010) might have contributed to conflicting behavioral test results in previous attentional studies. Most behavioral studies relied on verbal or motor responses without systematically addressing the motor bias, making the assessment of a solitary attentional dysfunction independent of motor dysfunction challenging.

Motor impairments and their influences on behavioral tests have long been known in the field of neurodegenerative diseases such as Parkinson's disease. Brown and Marsden (1991) demonstrated that cognitive slowing in Parkinson's disease can be misinterpreted as a primary executive deficit if motor demands are not systematically controlled (Brown and Marsden 1991). By using a dual‐task paradigm and comparing motor (tapping) and cognitive (random number generation) loads, they showed that only cognitively demanding secondary tasks selectively impaired Stroop task performance in Parkinson's patients. This emphasizes the need for motor bias correction in the interpretation of reaction time‐based cognitive tasks in neurodegenerative populations with known motor impairments.

Interestingly, Mohammadi et al. (2015) indicated longer reaction times in ALS patients (Mohammadi et al. 2015). In contrast to other studies, this study used a stop‐signal task in which patients and healthy controls were shown arrows to which they had to react by inhibiting their motor response (stop‐trial). These results were underlined in the accompanying fMRI section by parallel hyperactivation in the frontal inhibition networks (pre‐SMA, right inferior frontal gyrus). The hyper activation was caused by a mixture of activity in additional brain regions in the contralateral hemisphere compared to controls and stronger activation of brain regions (e.g., the frontal gyrus) compared to controls. While patients and controls had similar levels of accuracy and comparable reaction times, no analyses were performed whether hyper activation improved the behavioral response of ALS patients, which would have indicated a compensatory mechanism. Furthermore, neither behavioral nor fMRI analysis did not entail demographical and clinical variables, which might have significantly influenced the results.

Besides motor bias, other patient‐specific characteristics have been shown to contribute to behavioral test results, especially during the examination of neuropsychological function. In the study by Kobeleva et al. (2021), the behavioral performance of ALS patients was assessed using a verbal n‐back task with different amounts of difficulty (0‐back and 2‐back) (Kobeleva et al. 2021). Here, accuracy was significantly lower in older participants and reaction times were moderated by sex, but no further analyses were performed to explore the effects of sex and age in greater detail. These results highlight the importance of considering demographical variables when interpreting cognitive task performance in neurodegenerative populations. Besides age and gender, it is widely known that education compensates for disease‐related cognitive dysfunction (i.e., cognitive reserve).

To address motor limitations and the influence of demographical variables on attentional‐executive function in ALS, we chose the (modified) attention network test (ANT) (Firbank et al. 2016) with additional levels of task difficulty, adapted from the original task by Fan (Fan et al. 2002). The ANT is a visual, non‐verbal task that requires the participants to indicate the direction of the majority of arrows presented on a screen that can be preceded by a visual cue. As the scores are calculated by reaction time (RT) differences between stimulus combinations with varying difficulties, they are independent of the average motor speed and thus suitable for motor‐impaired patients. Furthermore, it is possible to adapt the level of difficulty of the executive task for further exploration of the effects of executive function on attentional performance. We specifically focused on potential influences of clinical and demographical variables on behavioral performance, instead of just regressing them out. Our study aimed to contribute quantitative evidence on whether attentional‐executive deficits in ALS patients without reported symptoms of cognitive dysfunction are early and independent features of the disease or are due to other processes such as aging. Our study results have implications for researchers focusing on testing cognition in ALS and other neurodegenerative disorders as well as for clinicians informing patients affected by ALS about possible cognitive impairments.

## 2 Methods

### 2.1 Participants

27 ALS patients were recruited for the study from the in‐ and outpatients’ clinics of the Center for Neurology of the University Hospital Bonn. They did not have any clinical signs of other current neuropsychiatric disorders and did not meet the criteria for front temporal dementia, based on ALS‐FTD classification criteria (Rascovsky et al. 2011). The diagnosis of ALS had been made by a board‐certified neurologist according to the El Escorial criteria (i.e., diagnosis of possible, probable, laboratory supported‐probable, or definite ALS (Brooks et al. 2000)). Among the ALS patients, 19 had a primary spinal onset (70%), and 8 had a primary bulbar onset (30%).

In order to perform the behavioral task, we only included patients who were able to use the index and middle fingers of their right hand to press buttons on a keyboard. The severity of functional impairment was assessed with the revised version of the ALS functional rating scale (ALSFRS‐R, (Cedarbaum et al. 1999)) and neurological examination.

We recruited 26 healthy controls who were matched for age, sex, and years of education and did not have any history of neurological or psychiatric disorder. All participants gave written informed consent to participate in the study. Due to technical difficulties, the neuropsychological performance task of one ALS patient could not be analyzed. The study was approved by the local ethics committee.

### 2.2 Behavioral Task

**FIGURE 1:**
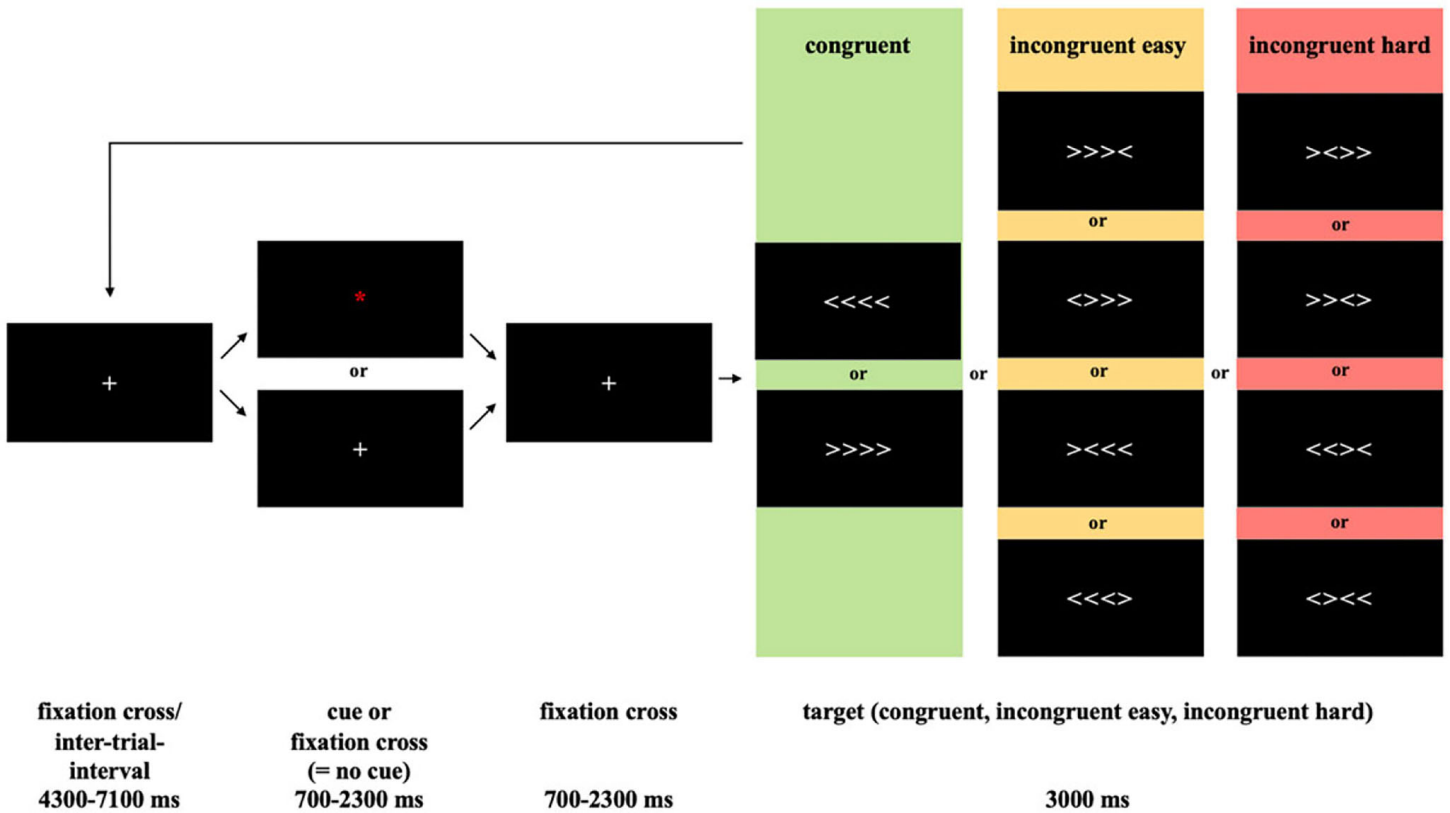
Task design for modified version of the attentional network test (ANT). A centered fixation cross is displayed, followed by either a fixation cross or a centered red cue and the fixation cross again. A variant of the three difficulties of stimuli (congruent, incongruent easy and incongruent hard) is then shown. The participants were required to press a button depending on the direction of the majority of the displayed arrows.

In a modified version of the ANT, adapted from (Firbank et al. 2016), our study participants were shown four arrowheads pointing to the left or right (see Figure 1). They were instructed to press the button with the index finger (left) or the middle finger (right) of their right hand in the direction of the majority of arrowheads.

Either all arrows were pointing in the same direction (congruent condition) or one was pointing in the opposite direction (incongruent condition). As an adjustment to the original ANT (Fan et al. 2002), the incongruent condition was graduated into two levels of difficulty: an opposite arrowhead at the beginning or end (easy incongruent condition) or an opposite arrowhead in the middle (hard incongruent condition) (Figure 1). Patients were instructed that a red asterisk would appear randomly between arrow tasks to alert them to the upcoming arrow task (alerting condition). Before starting the trial, all participants performed a short practice trial. Due to our focus on attentional‐executive function, we omitted the orienting condition (i.e., differing cue locations) from our study.

Our participants underwent three runs consisting of 48 trials each (except for one participant with only two trials due to technical difficulties). Altogether, the trial order was randomly assigned with a 50% probability of a congruent trial, 25% probability of an incongruent easy trial and 25% probability of an incongruent hard trial. A centered fixation cross was displayed for a duration of 4300–7100 ms, followed by either a fixation cross or a centered red cue for 700–2300 ms. Again, the fixation cross was displayed for 700–2300 ms. The flanker task was then displayed for 3000 ms or until a corresponding button was pressed.

To rule out the influence of motor impairment on reaction times, we considered relative mean reaction times in addition to absolute reaction times. For the relative reaction times, we examined effects that are linked to the activation of the frontoparietal brain network: alerting effect (mean reaction time of no cue trials minus mean reaction time of cue trials), executive effect (mean reaction time of all incongruent trials minus congruent trials) and conflict effect (mean reaction times of incongruent hard trials minus mean reaction times of incongruent easy trials). Percentages of relative reaction times were calculated for each effect: alerting effect in percent (mean reaction time of no cue trials minus mean reaction time of cue trials divided by the mean reaction time of no cue trials), executive effect in percent (mean reaction time of all incongruent trials minus congruent trials divided by mean reaction time of all incongruent trials) and conflict effect (mean reaction times of incongruent hard trials minus mean reaction times of incongruent easy trials divided by mean reaction times of incongruent hard trials).

### 2.3 Neuropsychological Testing

For neuropsychological testing, our participants underwent the cognitive part of the Edinburgh cognitive and behavioural ALS screen (ECAS, German version) (Abrahams et al. 2014). The assessment encompasses tests of ALS‐specific (language, verbal fluency, and executive function) and non‐specific cognitive domains (memory and visuospatial function), which can also be performed in the presence of motor or bulbar dysfunction. The ECAS was also used to distinguish our group into cognitively impaired and non‐impaired patients using the revised Strong criteria (Strong et al. 2017): ALS‐ci (cognitively impaired in at least one ALS‐specific cognitive domain) and ALS‐ni (no cognitive impairment). To do so, minimum scores were defined as cut‐off values of the German ECAS. (Abrahams et al. 2014)

### 2.4 Analysis of Behavioral and Neuropsychological Data

#### 2.4.1 Main Effects and Error Rates of Congruent, Incongruent Easy, and Hard Targets

Statistical analysis was conducted with Jamovi 2.6.6 (The Jamovi Project 2025). The behavioral data were analyzed with a repeated‐measures ANOVA with a between‐subject factor “group” (ALS vs. controls) and within‐subject factors of either “target” (i.e., different accuracies in reaction to congruent, incongruent‐easy and incongruent‐hard targets, or differences in reaction time differences indicating alerting, executive, and conflict effects). Age, sex, and years of education were included as covariates. Using an additional repeated‐measures ANOVA, we examined the influence of ALS‐specific domains (disease duration, disease progression, ALSFRS‐R, and executive function) as covariates. If significant effects were found in the ANOVA, we conducted post‐hoc tests with t‐tests or linear regressions to quantify the effects further. Due to the absence of significant results in ALS‐specific neuropsychological domains and the small group sizes of ALS‐ci (*n* = 6)/ALS‐ni (*n* = 21), we did not further compare cognitive subgroups. Effect sizes are reported as partial *η* ^2^ for ANOVAs and Cohen's d for *t*‐tests.

#### 2.4.2 Sub‐Analyses Cue on Target

Further analyses investigated the influence of cues on the executive and conflict effects. For both effects, we calculated the relative reaction times specific to each effect and expressed these as percentages (reaction time for the executive or conflict cued, divided by the reaction time for the executive or conflict uncued condition). We calculated a repeated‐measures ANOVA with a between‐subject factor “group” (ALS vs. controls) and a within‐subject factor “cue on target” (executive or conflict effect). Age, sex, and years of education were included as covariates. Due to the absence of significant results, post‐hoc tests were not performed.

## 3 Results

### 3.1 Clinical and Demographical Information

The demographics and clinical descriptions of the ALS and control groups can be found in Table 1. There were no significant differences in demographical variables between ALS patients and controls (all *p* > 0.05). Information on cognitive impairment follows below.

**TABLE 1:** Demographics, clinical variables and neuropsychological performance in ALS patients and controls. Means and standard deviations of ALS patients and controls in demographics (age, education, disease duration of ALS patients), clinical variables (ALS‐FRSr and progression rates of ALS patients) and results of the ECAS. No significant differences between groups regarding demographics or neuropsychology were found.

—	ALS (*n* = 27)		Controls (*n* = 26)		*p*‐values	Cohen's d
—	Mean	SD	Mean	SD	—	—
**Demographics**						
**Age**	58.70	8.98	58.0	7.31	0.769	−0.08
**Education [years]**	14.89	3.18	16.20	2.87	0.124	0.43
**Clinical variables**						
**ALS‐FRS‐R**	36.42	5.84	—	—	—	—
**Disease duration [months]**	48.59	85.78	—	—	—	—
**Progression rates [48‐ALS‐FRS/duration in months]**	0.39	0.30	—	—	—	—
**Neuropsychology** ^a^						
**Memory (max 24)**	16.78^a^	4.13^a^	17.11	2.83	0.451	0.21
**Visuospatial function (max 12)**	11.59^a^	0.84^a^	11.89	0.42	0.194	0.36
**ALS non‐specific score (max 36)**	28.37^a^	4.13^a^	29.00	2.89	0.305	0.28
**Language (max 28)**	26.78^a^	2.04^a^	27.50	0.84	0.203	0.35
**Verbal fluency (max 24)**	19.63^a^	3.04^a^	20.50	2.22	0.164	0.39
**Executive function (max 48)**	37.93^a^	5.69^a^	38.07	5.84	0.620	0.14
**ALS specific score (max 100)**	84.78^a^	8.22^a^	86.11	7.49	0.329	0.27
**Total score (max 136)**	113.15^a^	11.35^a^	115.11	8.30	0.301	0.29

*Note*: ^a^ALS patients *n* = 26.

### 3.2 Neuropsychological Testing

There were no significant differences in neuropsychological performance between the both groups (all *p* > 0.05, Cohen's *d* < 0,4), neither in total scores nor in the subscores (see Table 1). Six ALS patients met the criteria for cognitive impairment based on the revised strong criteria (ALS‐ci), and the other 21 patients were classified as ALS without cognitive impairment (ALS‐ni) (Stoppel et al. 2014). Detailed descriptions of ALS‐ni and ALS‐ci patients can be found in Table S1.

### 3.3 Attention Network Test (ANT)

#### 3.3.1 Main Effects

Table 2 summarizes the task accuracy and absolute reaction times.

**TABLE 2:** Error rates and mean reaction times of the ANT in ALS patients and controls. Means and standard deviations (SD) of error rates and reaction times of ALS patients and controls. ALS patients show significantly higher error rates in hard trials compared to healthy controls.

—	ALS (*n* = 27)		Controls (*n* = 26)		*p*‐values	Cohen's d
—	Mean	SD	Mean	SD	—	—
**Hits in percent**						
**Congruent**	99.8	0.74	100	0	0.109	0.45
**Incongruent easy**	97.6	3.25	98.3	1.84	0.351	0.26
**Incongruent hard**	96.3	5.22	99.0	1.57	0.014	0.70
**All**	98.1	2.23	99.2	0.81	0.022	0.65
**Absolute reaction times in milliseconds**						
**All**	967.22	258.11	868.47	260.64	0.172	−0.38
**Relative reaction times (effects) in percent**						
**Alerting**	2.32	7.51	1.70	8.87	0.783	−0.08
**Executive**	34.60	10.37	36.48	10.02	0.504	0.19
**Conflict**	9.11	10.70	12.78	10.27	0.210	0.35

General task performance: Although absolute reaction times were in general longer in ALS patients (mean reaction time ALS: 967.22 ms with a standard deviation of 258.11 ms; mean reaction time controls: 868.47 ms with a standard deviation of 260.64 ms), there was no significant group difference in reaction times (*p* > 0.127, Cohen's *d* = −0.38). Participants responded to an average of 98.7% of all trials (ALS: 98.1%, HC: 99,2%) with a minimum of 91.7% of correct responses (ALS: 91.7%, HC: 97.2%).

**FIGURE 2:**
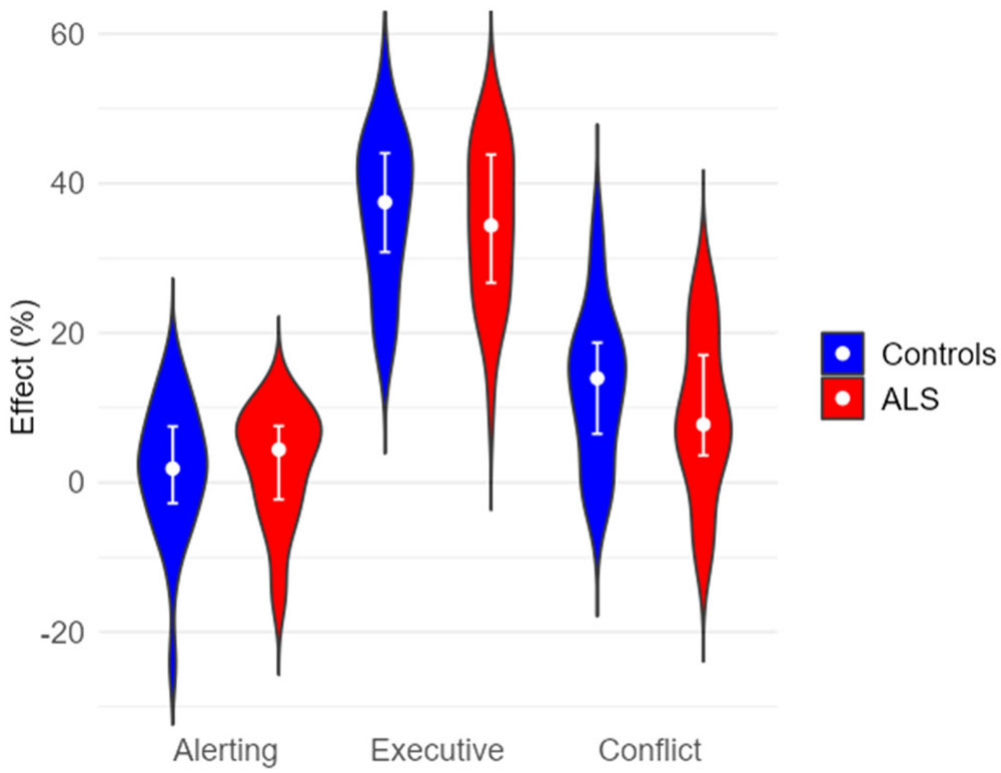
Alerting, executive, and conflict effect of the attention network test in ALS patients and controls. Relative reaction times of ALS patients (red) and healthy controls (blue) are presented in percent of difference of reaction times. While the alerting effect was not significant we found intact executive and conflict effects in both groups, with differences between ALS patients and controls.

Reaction time differences (i.e., alerting, executive, and conflict effects): Alerting, executive and conflict effects of ALS patients and controls are visualized in Figure 2. The repeated‐measures ANOVA showed a significant effect of target (*p* < 0.0001, partial η^2^ = 0.250) on reaction time differences, but it revealed no significant effect of target x group (*p* > 0.05, partial *η* ^2^ = 0.014). Post‐hoc *t*‐tests of reaction time differences showed that both groups showed intact executive and conflict effects (both *p*‐values < 0.001, Cohen's *d* = 3.501 for executive effect and 1.034 for conflict effect), while the alerting effect failed to reach significance (*p* = 0.077, Cohen's *d* = 0.248).

Independently of group membership, we found a significant interaction of target × education (*p* < 0.001, partial *η* ^2^ = 0.169). Using linear regression, we found that this significance was driven by executive effect (ß = 0.0126, *p* = 0.005), with higher RT differences in participants with less years of education. No significance was found for linear regressions of education on alerting and the conflict effects (*p*‐values > 0.099) (see Figure S1). The other covariates (sex, age) and interactions of covariates with group and target did not have a significant influence in the repeated‐measures ANOVA (*p*‐values > 0.190).

**FIGURE 3:**
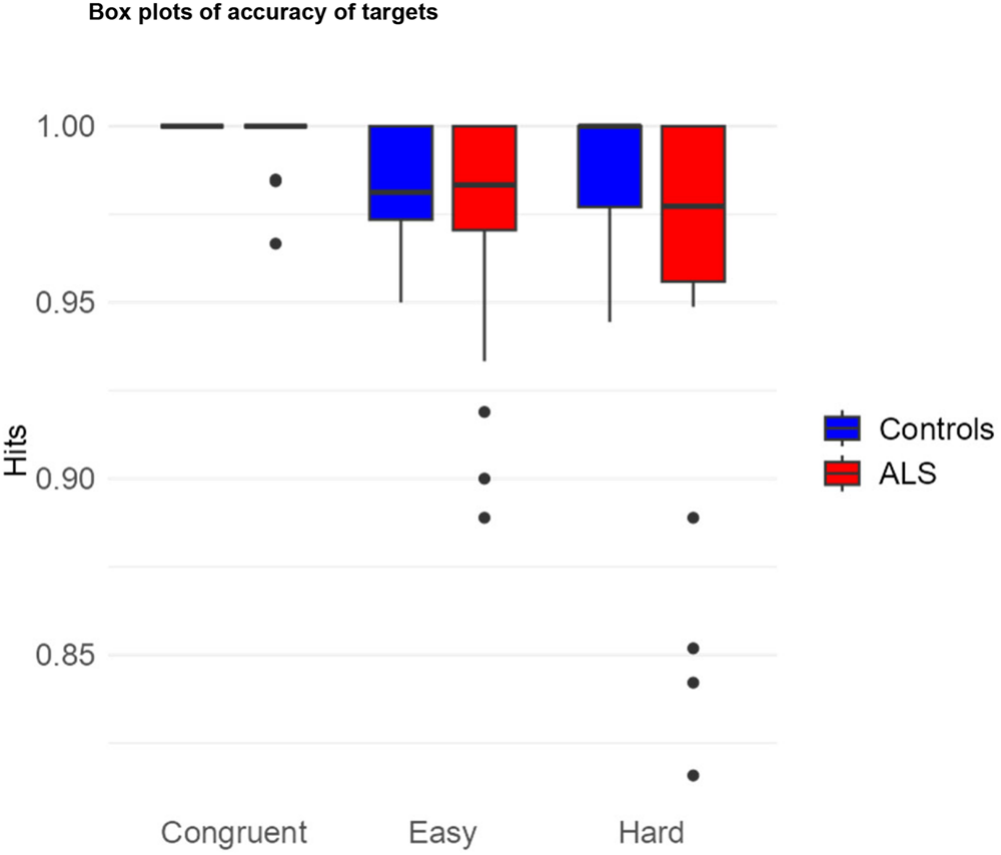
Task accuracy of three levels of difficulties of the attentional network test in ALS patients and controls. Task accuracy of congruent, incongruent easy, and incongruent hard trials in ALS patients (red) and controls (blue) are presented in percent. We found lower accuracy with increasing difficulty in ALS patients compared to controls.

**FIGURE 4:**
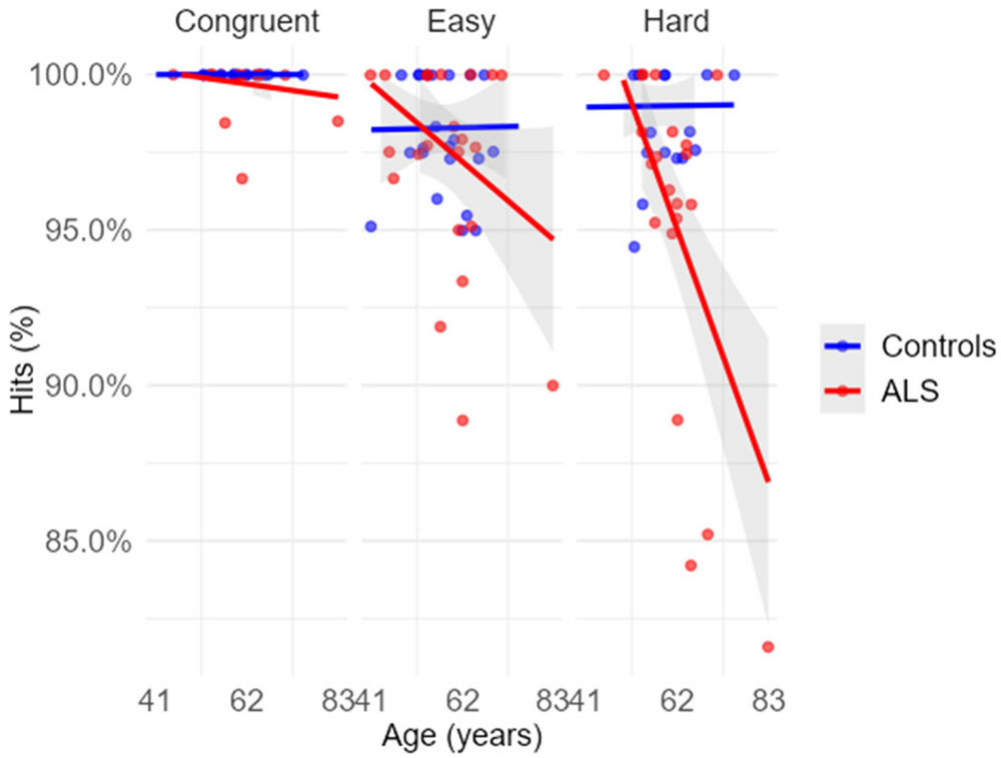
Effect of age on task accuracy. Task accuracy of ALS patients (red) and controls (blue) in different age range (range 41–83) at three difficulty levels (congruent, easy, difficult) are shown as a percentage. With increasing age and level of difficulty accuracy decreases in ALS patients.

Target accuracy: Task accuracy of ALS patients and controls across three levels of difficulty are shown in Figure 3. Repeated‐measures ANOVA of accuracies showed a significant effect of target x group (*p* = 0.036, partial *η* ^2^ = 0.071), target × age (*p* = 0.001, partial *η* ^2^ = 0.138), target × age × group (*p* = 0.002, partial *η* ^2^ = 0.129). All other covariates and their interactions, especially of age, group, and education, were non‐significant (with *p*‐values > 0.058 for the main effect of target and other *p*‐values > 0.086). To further explore the interaction between age, group, and target difficulty, we computed a linear regression of age on accuracy in different target difficulties and in both groups. Here, we found that age negatively influenced accuracy of the hard condition in ALS only (ß = −0.000241, *p* < 0.001, *R* ^2^ = 0.238). Other difficulty levels in ALS and all difficulty levels in controls did not reach significance (*p*‐values > 0.093). This relationship between group, age, and target is visualized in Figure 4 and the same pattern remains after removing ALS‐ci from the analysis (Figure S4), although we refrain from performing statistics due to the reduced sample size.

Effects of neuropsychological and clinical variables in ALS: Reaction time differences and accuracies of ALS patients showed no significant interaction with neuropsychological domains (executive function), or ALS‐specific variables (ALSFRS‐R, disease duration, and progression rate) (*p* > 0.457, partial *η* ^2^ < 0.039). Due to a small sample size of ALS‐ci with only *n* = 6, we did not perform subgroup analyses of ALS‐ni vs. ALS‐ci. However, we plotted ALS‐ni and ALS‐ni subgroups onto the main effects on RT differences and accuracies in Figures S2 and S3. Here, no clear clustering of ALS‐ci could be shown.

#### 3.3.2 Sub‐Analysis of Cue on Target

There was no significant impact of cues or difficulties on the effects in any group (all *p* > 0.05).

## 4 Discussion

In our study we aimed to examine attentional‐executive function in ALS without clinical symptoms suggestive of cognitive dysfunction, while controlling for effects of ALS‐induced motor impairment by examining relative reaction times using a modified version of the ANT (Firbank et al. 2016). Consistent with a motor‐adjusted version of the ECAS sub‐task, all participating ALS patients were able to complete three runs of the task without reporting severe fatigue effects (such as diminishing task accuracy). Our findings demonstrate the task's suitability for quantifying attentional and executive function in motor neuron disease with and without cognitive impairment across varying levels of disease severity. Moreover, the task proved feasible for even more severely motor‐impaired ALS patients with ALSFRS‐R scores as low as 23/48 points, provided that they were able to sit upright and press keyboard buttons with two fingers.

Reaction time differences were comparable to controls, demonstrating the task's ability to correct for motor bias. When examining relative reaction times, both groups exhibited intact executive and conflict effects, with no differences compared to controls. This finding is in contrast to studies focusing on classic paradigms without motor bias correction (Thorns et al. 2010). Notably, in neither group we were able to observe a significant alerting effect. This is consistent with our previous task implementation in patients with dementia (Firbank et al. 2016). Therefore, we hypothesize that the absence of an alerting effect might reflect an impact of age on alerting networks (Jennings et al. 2007; Mahoney et al. 2010) or inadequate salience or timing of the cues.

Although ALS patients had comparable task accuracies when reacting to congruent and incongruent easy targets, their task accuracy was significantly lower in the incongruent hard target compared to controls. This suggests that ALS patients are more error‐prone at higher task levels with increasing stimulus conflict but not in general. Previous studies investigating attentional and executive function did not employ tasks with varying levels of task difficulty and mostly reported no differences in accuracy (Mohammadi et al. 2015; Goldstein et al. 2011; McMackin et al. 2021). We hypothesize that using behavioral tasks incorporating different levels of difficulty may have been more sensitive in detecting subtle decreases in accuracy attributable to ALS pathology in non‐motor brain regions.

While previous studies on cognitive function largely included demographical influences on cognitive function as covariates of no interest in their analysis (Stoppel et al. 2014; McMackin et al. 2021; McMackin et al. 2019; Evdokimidis et al. 2002), we specifically investigated the influence of clinical and demographical factors on behavioral performance to disentangle the effect of ALS vs. aging‐related changes in attentional processing. We found that task accuracy deteriorated with increasing age in both groups. The aging‐related decline in accuracy was more pronounced in ALS, particularly when task difficulty and conflict were increased. Our results indicate a significant interaction between aging and ALS pathology, suggesting a potential breakdown of cognitive resources under high levels of difficulty and conflict. Potential contributing factors might include general aging‐related decline in attentional processing, a higher frequency of subtle aging‐related neurodegenerative processes (such as subclinical Alzheimer pathology), and vascular pathologies that were not controlled for in our study. While it is hard to establish a precise age cut‐off, our study data suggest that targeted cognitive screening might be especially beneficial for ALS patients above 60 years, even without clinical evidence of cognitive impairment. As aging‐related deterioration of accuracy was observed in both ALS‐ni and ALS‐ci patients, we hypothesize that this finding may reflect subclinical executive vulnerability in ALS patients, which lies below the ECAS acquisition threshold or might be only detectable with more sensitive tests than the attentional‐executive tests used in the ECAS. However, due to the small sample size of ALS‐ci patients in our study, our results regarding ALS‐ci should be cautiously interpreted, and studies with a larger cohort of ALS‐ci patients might have sufficient power to uncover attentional deficits in ALS‐ci vs. ALS‐ni that we did not detect.

Contrary to initial expectations and findings from literature on education moderating cognitive function in aging and neurodegenerative diseases (Kwak et al. 2020; Wilson et al. 2019; Kim et al. 2024; Fernández‐Rodríguez et al. 2024), we did not observe an effect of education on accuracy. Participants with higher levels of education showed smaller differences in reaction times under conditions with higher demands on cognitive control, as previously demonstrated (Tun and Lachman 2008). The effect of education on executive function suggests a possible compensatory effect of cognitive reserve, but we failed to find an ALS‐specific attenuating effect of education on task performance. While education was mostly considered in similar behavioral studies of cognition in ALS patients by matching patients with controls with similar educational background (Ringholz et al. 2005; Castelnovo et al. 2020; Evdokimidis et al. 2002), the body of literature with specific examinations of cognitive reserve in ALS is small. Our findings are consistent with a previous study by (Temp et al. 2022) that showed that cognitive domains such as attention were not protected by cognitive reserve. Notably, a study by Rhodes et al. (2024) showed the beneficial effect of complex occupational activity on cognitive reserve in ALS, which could be assessed in future behavioral studies as a potentially more informative proxy of cognitive reserve (Rhodes et al. 2024).

In summary, our study provides evidence that, in general, attentional‐executive function is unimpaired in ALS patients without clinical symptoms suggestive of cognitive impairment, even if the ECAS screening detected signs of subclinical cognitive dysfunction in about 25% of the tested patients. Despite comparable reaction times and accuracy rates in the overall ALS cohort, additional influences such as aging can lead to a faster breakdown of cognitive resources available for attentional processing. We acknowledge several limitations of our study that might have influenced our results and could be addressed in future studies. First, we did not account for genetic ALS variants in our analysis. Given that certain variants, such as c9orf72 mutations, are associated with an increased risk for cognitive impairment (Byrne et al. 2012), study participants with these mutations could have influenced our study results. Furthermore, due to the rarity and severity of the disease, we were only able to recruit a relatively small sample size, precluding further analysis of specific disease subgroups such as ALS‐ci vs. ALS‐ni comparisons. While in our cohort we did not find a visual clustering of ALS‐ci patients pointing towards general worse performance of ALS‐ci patients compared to ALS‐ni patients in the ANT, we did not reach a sufficient sample size of ALS‐ci patients to perform statistical analyses of ALS‐ni vs. ALS‐ci subgroups. Additionally, certain ALS subgroups with more severe motor impairment and resulting inability to participate in the task had to be excluded, limiting the generalizability of results in cognition on the entire ALS population. Here, studies employing eye‐tracking methodologies for response tracking might prove useful for including more severely impaired patients (Schmitz‐Peiffer et al. 2022).

Our study highlights the importance of quantifying and understanding potential demographical influences and co‐pathologies in studies of cognition in neurodegenerative disorders. Specifically, we advocate for employing task designs that account for motor bias in neurodegenerative disorders such as ALS. Furthermore, our study findings suggest that aging acts as a negative moderating factor in studies of cognition, potentially leading to a premature depletion of neural resources in the presence of ALS‐related pathologies or other neurodegenerative processes. On a positive note for clinical practice, our results may inform individuals affected by ALS about potential cognitive impairments. We would like to emphasize that clinical practitioners could reassure patients with ALS that attentional‐executive function is often preserved in young individuals with sporadic ALS.

## Author Contributions

**Maher Zoubi**: investigation, writing – original draft, methodology, visualization, writing – review & editing, formal analysis, project administration, data curation, **Patrick Weydt**: data curation, conceptualization, writing – review & editing, supervision, resources, **Xenia Kobeleva**: conceptualization, investigation, writing – original draft, writing – review & editing, visualization, formal analysis, project administration, data curation, supervision, resources, validation, methodology

## Funding

The authors have nothing to report.

## Ethics Statement

The study was approved by the local ethics committee.

## Conflicts of Interest

The authors declare no conflicts of interest.

## Supporting information




**Figure S1** Effect of education on task effects.
**Figure S2** Alerting, executive, and conflict effect of the attention network test in ALS‐ni and ALS‐ci patients.
**Figure S3** Task accuracy of three levels of difficulties of the attention network test in ALS‐ni and ALS‐ci patients.
**Figure S4** Effect of age on task accuracy in ALS‐ni vs. controls.
**Table S1** Demographics, clinical variables, and neuropsychological performance in ALS‐ni and ALS‐ci patients.

## Data Availability

Group data that support the findings of this study are available on request from the corresponding author.
