# Supplementary material for: Attentional Function in Patients With Amyotrophic Lateral Sclerosis is Moderated by Age and Education
Source: Brain Behav. 2026 Jun 3;16(6):e71511. doi: 10.1002/brb3.71511 (PMC13580763; doi:10.1002/brb3.71511)
Supplement: Supplementary file 1 [file BRB3-16-e71511-s001.doc]

## Supplementary

**Figure S1. Effect of education on task effects.** Relative reaction times of ALS patients (red) and healthy controls (blue) with different years of education are presented in percent. The executive effect (difference between congruent and incongruent) depends on years of education, suggesting effects of cognitive reserve.

**Figure S2. Alerting, Executive, and Conflict Effect of the Attention Network Test in ALS-ni and ALS-ci patients.** Relative reaction times of ALS-ni patients (orange) and ALS-ci patients (purple) are presented in percent of difference of reaction times.

**Figure S3. Task accuracy of three levels of difficulties of the Attentional Network Test in ALS-ni and ALS-ci patients.** Task accuracy of congruent, incongruent easy, and incongruent hard trials in ALS-ni patients (orange) and ALS-ci (purple) are presented in percent.

**Figure S4. Effect of age on task accuracy in ALS-ni vs. controls.** Task accuracy of ALS-ni patients (red) and controls (blue) after removing ALS-ci patients in different age range (range 41-83) at three difficulty levels (congruent, easy, difficult) are shown as a percentage. With increasing age and level of difficulty accuracy decreases in ALS-ni patients.

|  | **ALS-ni (n = 21)** | | **ALS-ci (n = 6)** | |
| --- | --- | --- | --- | --- |
|  | **mean** | **SD** | **mean** | **SD** |
| **Age** | 57.14 | 8.10 | 64.17 | 10.53 |
| **Education [years]** | 14.69 | 3.02 | 15.58 | 3.93 |
| **ALS-FRS-R** | 39.33 | 6.01 | 34.50 | 4.51 |
| **Disease**  **duration [months]** | 53.16 | 99,48 | 30.00 | 16.56 |
| **Progression rates [48-ALS-FRS/duration in months]** | 0.326 | 0.255 | 0.627 | 0.33 |
| **Memory (max 24)** | 17.100 | 4.340 | 16.500 | 3.450 |
| **Visuospatial function (max 12)** | 11.700 | 0.657 | 11.667 | 0.816 |
| **ALS non-specific score (max 36)** | 28.800 | 4.262 | 28.167 | 2.858 |
| **Language (max 28)** | 27.400 | 1.046 | 26.000 | 1.789 |
| **Verbal fluency (max 24)** | 20.500 | 1.933 | 17.667 | 4.457 |
| **Executive function (max 48)** | 38.950 | 4.685 | 36.167 | 7.574 |
| **ALS specific score (max 100)** | 86.400 | 6.419 | 81.667 | 11.587 |
| **Total score  (max 136)** | 115.200 | 9.924 | 109.833 | 13.273 |

**Table S1. Demographics, clinical variables and neuropsychological performance in ALS-ni and ALS-ci patients.** Means and standard deviations of ALS-ni and ALS-ci patients a in demographics (age, education, disease duration of ALS patients), clinical variables (ALS-FRSr and progression rates of ALS Patients) and results of the ECAS. The dataset of one ALS patient is missing (n = 20 for the neuropsychology for ALS-ni).
